# Supplementary material for: The influence of time pressure on translation trainees’ performance: Testing the relationship between self-esteem, salivary cortisol and subjective stress response
Source: PLoS One. 2021 Sep 30;16(9):e0257727. doi: 10.1371/journal.pone.0257727 (PMC8483415; doi:10.1371/journal.pone.0257727)
Supplement: S1 File — (DOCX) [file pone.0257727.s001.docx]

Supporting information S1 File

# Three translation tasks

## Task 1 (150 words)

She made a noise. Could have been yes. Could have been no, but the blanket was pulled off her head and she gasped for air.

An arched hall stretched before her, lit with lamps. The thin hiss of gas. Plants everywhere, and the smell of carbolic soap. On the floor were tiles, reaching out in all directions, polished till they shone, some in the shapes of flowers, but the flowers were black. She knew then that this was no police station, and started shouting in fear, until a young woman in uniform appeared from the darkness and slapped her on the cheek.

Irish. Ella whipped her head back, tears in her eyes though she wasn’t crying. She knew those Irish girls. There were plenty at the mill. They were mean as hell.

Another woman came, and they put their hands beneath her armpits and began pulling her towards two doors.

## Task 2 (153 words)

The day was fair and warm. She walked slowly, careful on the rutted earth. On either side of the lane were meadows, and the meadows were filled with cattle, lazy in the sun. Summer flowers grew wild from the cracks in the tumbled stone walls. The land was green. Somewhere at the edge of things she could smell the sea.

Rounding a corner she saw the house: low and long, with three windows at the front. Whitewashed. A house that someone had taken care over. Around it lay a plot of land, where the tall stalks of vegetables grew in rows, ready to be gathered in. Close by stood a barn, where a man was working at the top of a ladder, the sound of his hammer bright on the air.

She stopped. Caught her breath. The man had his back to her, absorbed in his work. He had not seen her yet.

## Task 3 (153 words)

She looked down at herself. She had taken such care as she dressed this morning, but she was all wrong suddenly: her shoes too tight, the colour of her dress too loud. Her hat too smart for the warmth of the day. It was not too late to turn back. He would never know she had come.

She closed her eyes, the filtered light of the sun flickering against her lids. She had waited for this moment for too long.

The man’s hammer had stopped. She opened her eyes and the day burst in upon her.

The man had seen her. He was standing on the earth now, facing out, his gaze steady. She could not read his face. Her heart stalled.

She lifted her chin. Took a breath. He would not see her falter.

She walked towards the gate, and when she reached it, she opened her mouth and spoke his name.
